# The oncogenic functions of SPARCL1 in bladder cancer

**DOI:** 10.1111/jcmm.70196

**Published:** 2024-11-15

**Authors:** Changjiu Li, Hui Yuan, Jun Chen, Kun Shang, Huadong He

**Affiliations:** ^1^ Department of Urology, Affiliated Hangzhou First People's Hospital Westlake University School of Medicine Hangzhou China; ^2^ The Fourth Clinical Medical College Zhejiang Chinese Medical University Hangzhou China; ^3^ Department of Urology Ninghai First Hospital Ningbo

**Keywords:** bladder cancer, immune infiltration, M2 macrophage, prognosis, SPARCL1

## Abstract

Secreted protein, acidic and rich in cysteine‐like 1 (SPARCL1) belongs to the SPARC family of matricellular proteins. However, underlying functions of SPARCL1 in bladder cancer (BCa) remain understudied. We performed an integrated search for the expression patterns of SPARCL1 in relation to various clinicopathological features of BCa. We then carried out Gene Ontology (GO) enrichment analysis, Kyoto Encyclopedia of Genes and Genomes (KEGG) pathway enrichment analysis, and gene set enrichment analysis (GSEA). Furthermore, we investigated the correlations between SPARCL1 and immunological features, such as tumour mutation burden (TMB), immune activation processes, immune checkpoint expression, tumour immune dysfunction and exclusion (TIDE) scores, and chemotherapeutic sensitivity in BCa. Our analysis revealed that SPARCL1 was downregulated across multiple cancers. In BCa, elevated SPARCL1 was linked with advanced histopathologic stage, higher T and N stage, and poorer prognosis in the clinical cohort. In vitro experiments demonstrated that increased SPARCL1 expression inhibited cell proliferation, migration, and invasion. Additionally, highly expressed SPARCL1 was linked to elevated immune, stromal and ESTIMATE scores, as well as an increase in naive B cells, M2 macrophages, and resting mast cells. We observed a moderate correlation between SPARCL1 expression and CD163, VSIG4 and MS4A4A, which are markers of M2 macrophages. Furthermore, SPARCL1 expression was positively related to TMB, immune activation processes, TIDE scores, immune checkpoint expression, and chemotherapeutic sensitivity in BCa. Our study highlights the potential involvement of SPARCL1 in macrophage recruitment and polarization and suggests its utility as a biomarker for prognosis in BCa.

## 1 INTRODUCTION

Bladder cancer (BCa) remains a significant reason of cancer‐related deaths, estimated to have nearly 212,536 related deaths in 2020.^1^ The advent of immunotherapy has revolutionized BCa treatment, with the introduction of antibodies targeting programmed cell death protein‐1 (PD‐1), such as avelumab and pembrolizumab.^2^, ^3^ Whereas, only a few patients benefit from immunotherapy, and the current prognostic factors, including tumour mutation burden (TMB), microsatellite instability (MSI), and PD‐1/PD‐L1 expression, are insufficient for accurately predicting immunotherapeutic response.^4^, ^5^ To address this challenge, ongoing clinical trials are exploring more effective immune checkpoint blockade (ICB) strategies, and there is a growing need to understand the tumour microenvironment and identify reliable biomarkers.

One potential candidate is secreted protein, acidic and rich in cysteine‐like 1 (SPARCL1), which belongs to the SPARC family of matricellular proteins, along with SPARC/osteonectin, SPARC‐related modular calcium‐binding proteins, testicans and follistatin‐like protein.^6^ SPARCL1 has been implicated in essential functions such as cell adhesion and proliferation.^7^, ^8^, ^9^ For instance, it has been shown to induce CCL2 expression through binding to TLR4 and activating the NF‐κB/p65 pathway, thereby promoting non‐alcoholic steatohepatitis progression.^10^ Additionally, SPARCL1 has been found to stabilize vessel integrity and counteract inflammatory bowel disease.^11^ Notably, SPARCL1 is downregulated in various cancer types, including colorectal carcinoma and prostate carcinoma.^12^, ^13^, ^14^ Reduced expressed SPARCL1 has been related to favourable prognosis in colorectal cancer, while its upregulation in tumour vessel endothelial cells has been linked to better survival in colorectal carcinoma.^15^ In prostate cancer, SPARCL1 has been shown to suppress migration and invasiveness.^16^ However, the precise functional significance of SPARCL1 and its interactions with the tumour microenvironment remain unclear.

The advancements in next‐generation sequencing and whole exome sequencing have facilitated a better understanding of SPARCL1's role in BCa. We aimed to figure out the expression patterns of SPARCL1 using Cancer Genome Atlas (TCGA) database and comprehensively explored its associated signalling pathways. Additionally, we examined the association between SPARCL1 and immune microenvironment in BCa, with a specific focus on evaluating the prognostic potential of SPARCL1 as a biomarker for immunotherapy and chemotherapy. Ultimately, our findings aim to enhance the understanding of SPARCL1's functions, assist clinicians in selecting appropriate treatment options, and provide predictive insights into patient outcomes.

## 2 MATERIALS AND METHODS

### 2.1 Data collection

Normalized RNA‐seq data and clinical data for BCa patients were retrieved from the TCGA‐BLCA program. The gene expression data were normalized to the HTSeq‐FPKM format. The SPARCL1 protein expression statuses in BCa tissues were obtained from the Human Protein Atlas (https://www.proteinatlas.org/). The transcriptional patterns of SPARCL1 in various human cancer types were downloaded from TIMER2.0 database.^17^ All data collection procedures were carried out according to relevant regulations.

### 2.2 Assessment of clinicopathological features

The clinical data, including age, gender, histological grade, TNM (tumour, node, metastasis) status and survival were integrated with the normalized RNA‐seq data. BCa samples were grouped in high and low SPARCL1 subgroups, and SPARCL1 expression patterns at different clinicopathological features of BCa were analysed using the “limma” R package. Kaplan–Meier analysis was performed to assess the prognosis of BCa samples with different SPARCL1 statuses. ROC curves were drawn using “survivalROC” package.

### 2.3 Assessment of immunological features

The RNA‐seq information was utilized to analyse the tumour microenvironment with different SPARCL1 expression statuses. The ESTIMATE algorithm was employed to measure the stromal and immune infiltration of each BCa sample.^18^ Immune, stromal and ESTIMATE scores were calculated for every BCa patients from TCGA‐BLCA dataset.^18^ The CIBERSORT algorithm, which characterizes the cell composition from transcriptome profiles, was applied to determine the immunological composition in each BCa sample.^19^ The mRNA expression profiles of BCa samples were normalized using the “limma” R package. The gene signature file LM22, defining 22 immune cell types, was used for estimating immune cell composition of each BCa sample (https://cibersort.stanford.edu/). Successful deconvolution was considered when the *p*‐value was less than 0.05. The association between SPARCL1 expression and immune markers was evaluated using the TIMER website.^20^

### 2.4 Functional enrichment analysis

Differential expression genes were retrieved by the “limma” R package with a significance threshold of |log2FC| ≥ 1 and *p* < 0.001. The Entrez Gene IDs of these genes were obtained based on the genomewide annotation package. Gene Ontology (GO) and Kyoto Encyclopedia of Genes and Genomes (KEGG) analyses were performed using the “clusterProfiler” R package. Additionally, gene set enrichment analysis (GSEA) was conducted with criteria of *p* < 0.05 for comparing the differences in activated signalling pathways between different groups based on SPARCL1 expression.

### 2.5 Assessment of TMB, activities of immune activation processes, tumour immune dysfunction and exclusion and immune checkpoint expression

Various scores were calculated to predict the effectiveness of immunotherapeutic treatment in BCa patients. The TMB score was calculated as the total number of somatic nonsynonymous mutations, including base substitutions, deletions or insertions, normalized to the total number of metabases sequenced. The enrichment of 13 immune processes between the two subgroups was measured by Single Sample Gene Set Enrichment Analysis (ssGSEA). TIDE scores were evaluated based on the TIDE database (http://tide.dfci.harvard.edu). Immune checkpoint expression was assessed.

### 2.6 Assessment of drug sensitivity

The Genomics of Drug Sensitivity in Cancer (GDSC) project (https://www.cancerrxgene.org/) provided information on chemotherapeutic sensitivity for 138 types of anticancer drugs.^21^ The “pRRophetic” R package was adopted for computing the semi‐inhibitory concentration (IC50) of common chemotherapeutic drugs in the two different SPARCL1‐expressed subgroups.^22^

### 2.7 Patient tissue specimens

The BCa tissues and paired normal tissues were obtained from patients undergoing a surgical procedure at the Department of Urology of Hangzhou First People's Hospital (Hangzhou, China). In total, 20 pairs of tissue specimens were immediately frozen in liquid nitrogen after surgical removal and stored at −80°C. All the patients provided written consent, and the use of BCa tissues was approved by the Ethics Committee from Hangzhou First People's Hospital.

### 2.8 RNA isolation and quantitative RT‐PCR

Total RNA was isolated from cells using Trizol reagent (Takara, Dalian, China) following the provided protocols. The extracted RNA was used for cDNA synthesis by the Prime Script RT Master Mix (Takara, Japan). qPCR was adopted on a 7500 real‐time PCR system (Applied Biosystems, USA) following the provided instructions. The relative expression levels were calculated using the 2‐∆∆CT method, with β‐actin mRNA serving as the internal control. The primer sequences are listed in Table 1.

**TABLE 1:** The primer sequences.

Gene	Primer
SPARCL1	F: 5′‐GCCTGGAGAGCACCAAGAGGCC‐3′
	R: 5′‐ATGGTCCCCAGCCAAAAGCCTC‐3′
β‐Actin	F: 5′‐CACGATGGAGGGGCCGGACTCATC‐3′
	R: 5′‐TAAAGACCTCTATGCCAACACAGT‐3′

### 2.9 Cell culture and treatments

The T24 and EJ cells were purchased from the Shanghai Institute of Cell Biology, Chinese Academy of Sciences. They were cultured in RPMI‐1640 medium supplemented with 10% fetal bovine serum (FBS, Gibco, USA) and 1% penicillin/streptomycin (Gibco, USA). The cells were incubated in an incubation cabinet with 5% CO_2_.

### 2.10 Cell transfection

The human SPARCL1 cDNA was cloned into the pCDNA3.1 vector by Tsingke Biotechnology (Beijing, China) to create the SPARCL1 overexpression plasmid. Three small interfering RNAs (siRNAs) sequences were constructed by Tsingke Biotechnology (Beijing, China). Sequence of siRNAs were listed in Table 2. The transfection procedure was conducted using jetPRIME (Polyplus‐transfection, France) following the provided protocols. Total RNA was collected 48 h after transfection.

**TABLE 2:** siRNA sequences.

Gene	Sense	Anti‐sense
siSPARCL1‐1	5′‐GGAAGAGUGUGAUCAACCATT‐3′	5′‐UGGUUGSUCAGACUCUUCCTT‐3′
siSPARCL1‐2	5′‐GACAAUCAGACCUAUGCUATT‐3′	5′‐UAGCAUAGGUCUGAUUGUCTT‐3′
siSPARCL1‐3	5′‐GUUCCUUCACAGAUUCUAATT‐3′	5′‐UUAGAAUCUGUGAAGGAACTT‐3′

### 2.11 Cell proliferation, migration, invasion and wound healing assays

The proliferation of T24 and EJ cells was measured by the CCK‐8 kit (Yeasen, China) following the provided protocols. 2 × 10^3^ cells were plated into 96‐well plates. After 24 h, the cells were treated with 10 μL CCK‐8 reagent, and incubated in the dark for 2 h. The absorbance was measured at 450 nm.

Transwell assay (8 μm pore size, Corning) was adopted to evaluate the invasion and migration capacities of T24 and EJ cells. Approximately 4 × 104 T24 or EJ cells were suspended with 200 μL of serum‐free medium and inoculated in the upper chamber precoated with or without Matrigel (BD Science, USA) for invasion and migration assay. The bottom chamber was filled with medium containing 20% FBS. After incubation at 37°C for the appropriate time, the cells on the lower membrane surface were fixed with formaldehyde and stained with crystal violet for counting and photography.

Cells were seeded in a six‐well plate, and wounds were created using a 200 μL pipette tip. Photographs were taken after 24 or 36 h to measure the reduction in wound distance.

### 2.12 Immunohistochemistry analysis

In total, 26 BCa tissues and adjacent normal tissues were immunostained by anti‐SPARCL1, anti‐CD163, anti‐NOS2 and HRP‐conjugated goat anti‐rabbit IgG, respectively. Proteins expression levels were analysed using Image‐Pro Plus 4.5 software (Media Cybernetics). The expression of SPARCL1 was determined through H‐score.

### 2.13 Statistical analysis

Continuous variables such as TMB score, TIDE score and immune checkpoint expression were compared using the Wilcoxon rank‐sum test, while the chi‐squared test was performed for categorical variables. Kaplan–Meier curves with log‐rank tests were used to evaluate the overall survival (OS) of different groups. All statistical analyses were conducted using R software version 4.0.2. A *p*‐value less than 0.05 was considered statistically significant.

## 3 RESULTS

### 3.1 SPARCL1 expression landscape in pan‐cancer

**FIGURE 1:**
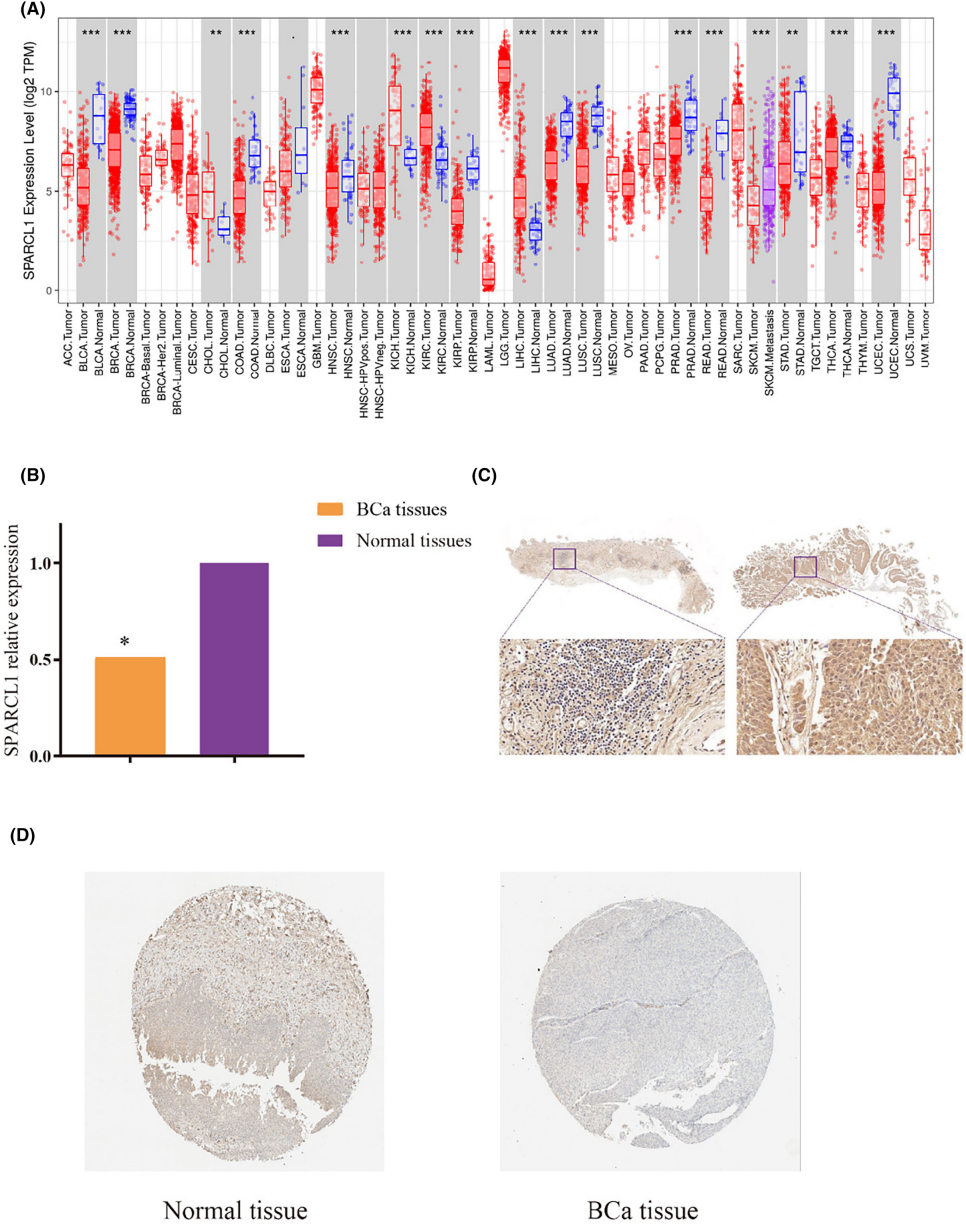
The landscape of SPARCL1 expression. (A) The aberrant transcriptional levels of SPARCL1 across cancers based on TIMER 2.0 database. (B) The expression level of SPARCL1 was downregulated in BCa tissues. (C) The protein level of SPARCL1 was inhibited in BCa tissues (left) and normal tissues (right). (D) The protein level of SPARCL1 was inhibited in BCa tissues and normal tissues. *p < 0.05 **p < 0.01 and ***p < 0.001.

Analysis of transcriptional features of SPARCL1 across various cancers was conducted using the TIMER2.0 website. The results, as depicted in Figure 1A, revealed widespread dysregulation of SPARCL1 in cancer tissues compared to normal tissue samples. Most cancers showed a significant decrease in SPARCL1 expression, including bladder urothelial carcinoma (BLCA), breast invasive carcinoma (BRCA), colon adenocarcinoma (COAD), head and neck squamous cell carcinoma (HNSC), kidney renal papillary cell carcinoma (KIRP), lung adenocarcinoma (LUAD), lung squamous cell carcinoma (LUSC), prostate adenocarcinoma (PRAD), rectum adenocarcinoma (READ), thyroid carcinoma (THCA), uterine corpus endometrial carcinoma (UCEC) (*p* < 0.001) and stomach adenocarcinoma (STAD) (*p* < 0.01). Conversely, SPARCL1 was found to be upregulated in kidney chromophobe (KICH), kidney renal clear cell carcinoma (KIRC), liver hepatocellular carcinoma (LIHC) (*p* < 0.001), and cholangiocarcinoma (CHOL) (*p* < 0.01). These findings indicate SPARCL1 may function in tumorigenesis across different types of cancer. To further investigate the expression of SPARCL1 in BCa, we detected the expression level of SPARCL1 in 20 BCa tissues and paired normal tissues, and the results confirmed that SPARCL1 had significant low expression in BCa tissues (Figure 1B).

Furthermore, an assessment of protein expression based on the Human Protein Atlas (HPA) database revealed a decreased expression of SPARCL1 in BCa tissues (Figure 1D). The immunohistochemistry (IHC) analysis was applied to assess the protein expression of SPARCL1 in BCa tissues. The protein expression of SPARCL1 was obviously decreased in BCa specimens compared to adjacent normal tissues (H‐score: 107.05 ± 30.28 vs. 151.5 ± 15.2, *p* < 0.01) (Figure 1C).

### 3.2 SPARCL1 expression was related to several clinicopathological features in BCa

**FIGURE 2:**
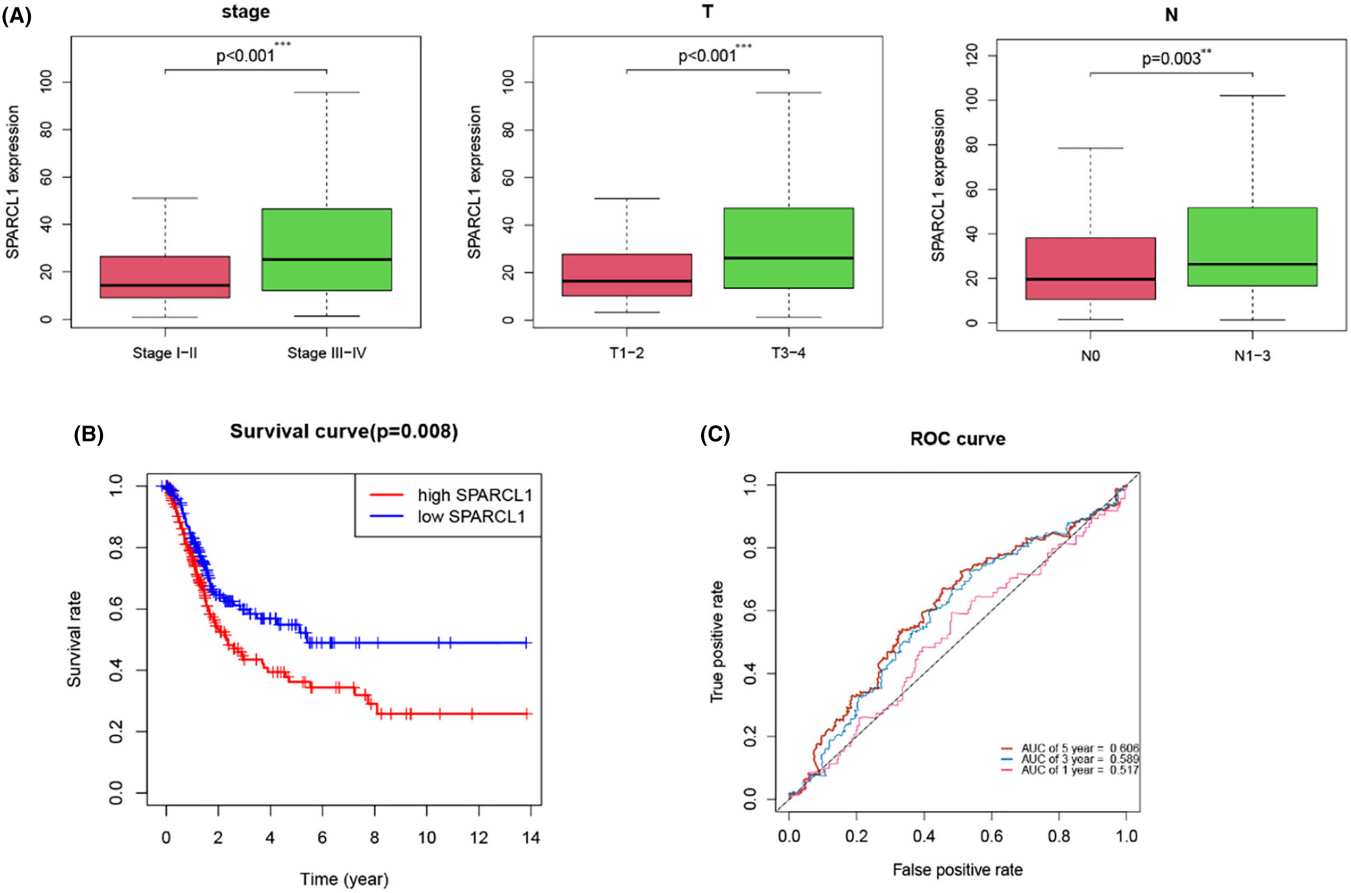
SPARCL1 expression was related to several clinicopathological features in BCa. (A) SPARCL1 expression levels were upregulated as the BCa stage increased. (B) Patients with highly expressed SPARCL1 were found to have worse OS. (C) The ROC curve of SPARCL1. **p < 0.01 and ***p < 0.001.

We proceeded to investigate the expression patterns of SPARCL1 in relation to various clinicopathological features of BCa and revealed a significant association between SPARCL1 and multiple clinicopathological stages in BCa. Notably, there were significant differences observed in the histopathologic stage (*p* < 0.001), T stage (*p* < 0.001) and N stage (*p* = 0.003) (Figure 2A). Our findings indicated that as the BCa stage increased, SPARCL1 expression levels were upregulated. Moreover, highly expressed SPARCL1 was related to an increased likelihood of higher T stage. Additionally, we confirmed a correlation between SPARCL1 expression and lymphatic metastasis. Patients with lymphatic metastasis exhibited upregulated SPARCL1 expression compared to those without metastasis. Furthermore, patients with highly expressed SPARCL1 were found to have worse OS (*p* = 0.008) (Figure 2B). The 5‐year relative survival rate for individuals with highly expressed SPARCL1 was 36.2% (95% CI: 0.278–0.471), whereas it was 52.2% (95% CI: 0.428–0.637) for the low SPARCL1 expression group. ROC curves were generated (Figure 2C). In our clinical cohorts, we verified there were significant differences observed in the T stage (*p* = 0.013), lymphatic metastasis (*p* = 0.045), and histological grade (*p* = 0.013) (Table 3; Figure S2). Collectively, these results indicate SPARCL1 might have an oncogenic function in BCa.

**TABLE 3:** The relationship between SPARCL1 protein expression and clinicopathological characteristics in BCa patients.

	SPARCL1 H‐score				*p*
	<60	60–120	120–225	≥225	
Gender					
Male	3	6	11	4	0.76
Female	0	0	2	0	
Age (years)					
<65	0	0	6	0	0.055
≥65	3	6	7	4	
Tumour sizes (cm)					
<3	2	4	10	1	0.872
≥3	1	2	3	3	
T stage					
T1‐2	3	2	1	1	0.013*
T3‐4	0	4	12	3	
Lymphatic metastasis					
No	0	4	3	1	0.045*
Yes	2	2	10	3	
Distant metastasis					
No	2	5	7	3	0.723
Yes	1	1	6	1	
Histological grade					
G1	1	2	0	0	0.013*
G2	1	0	0	0	
G3	1	4	13	4	

* *p* < 0.05.

### 3.3 Over‐expression of SPARCL1 inhibited cell proliferation, migration and invasion in vitro

**FIGURE 3:**
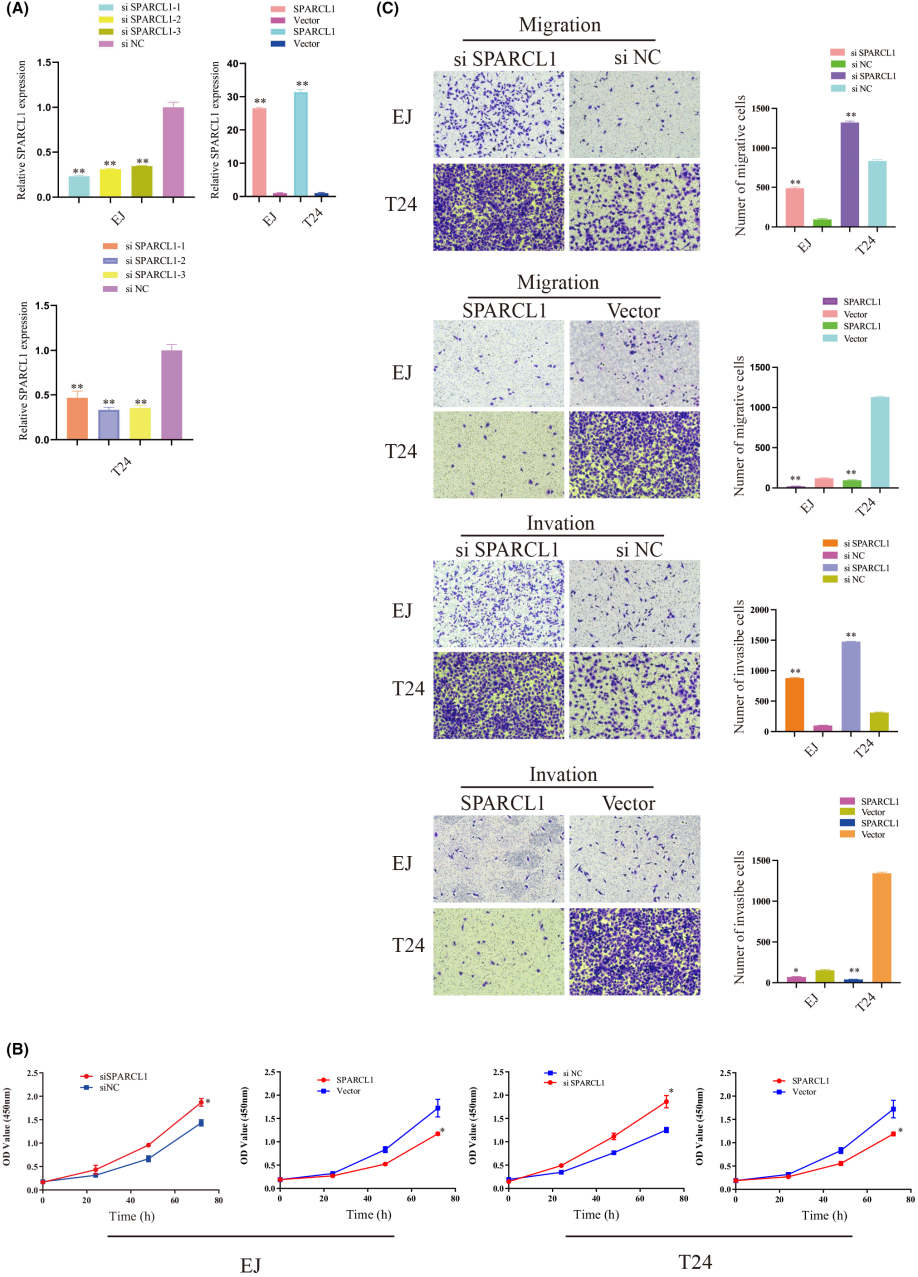
SPARCL1 was related to cell proliferation, migration and invasion in vitro. (A) qPCR analysis of the knockdown efficiency of siRNAs and the overexpression efficiency of plasmids against SPARCL1 in EJ and T24 cell lines. β‐Actin was used as an internal control. (B) CCK8 assay for the viability of EJ and T24 cells transfected with siSPARCL1, control siRNAs (siNC), SPARCL1 or control vector. (C) Cell migration and invasion abilities of EJ and T24 cells transfected with siSPARCL1, siNC, SPARCL1 or control vector were evaluated by transwell migration and invasion assays. Data are mean ± SD, *n* = 3. * indicates *p* < 0.05, ** indicates *p* < 0.01 in a two‐tailed Student's *t* test.

**FIGURE 4:**
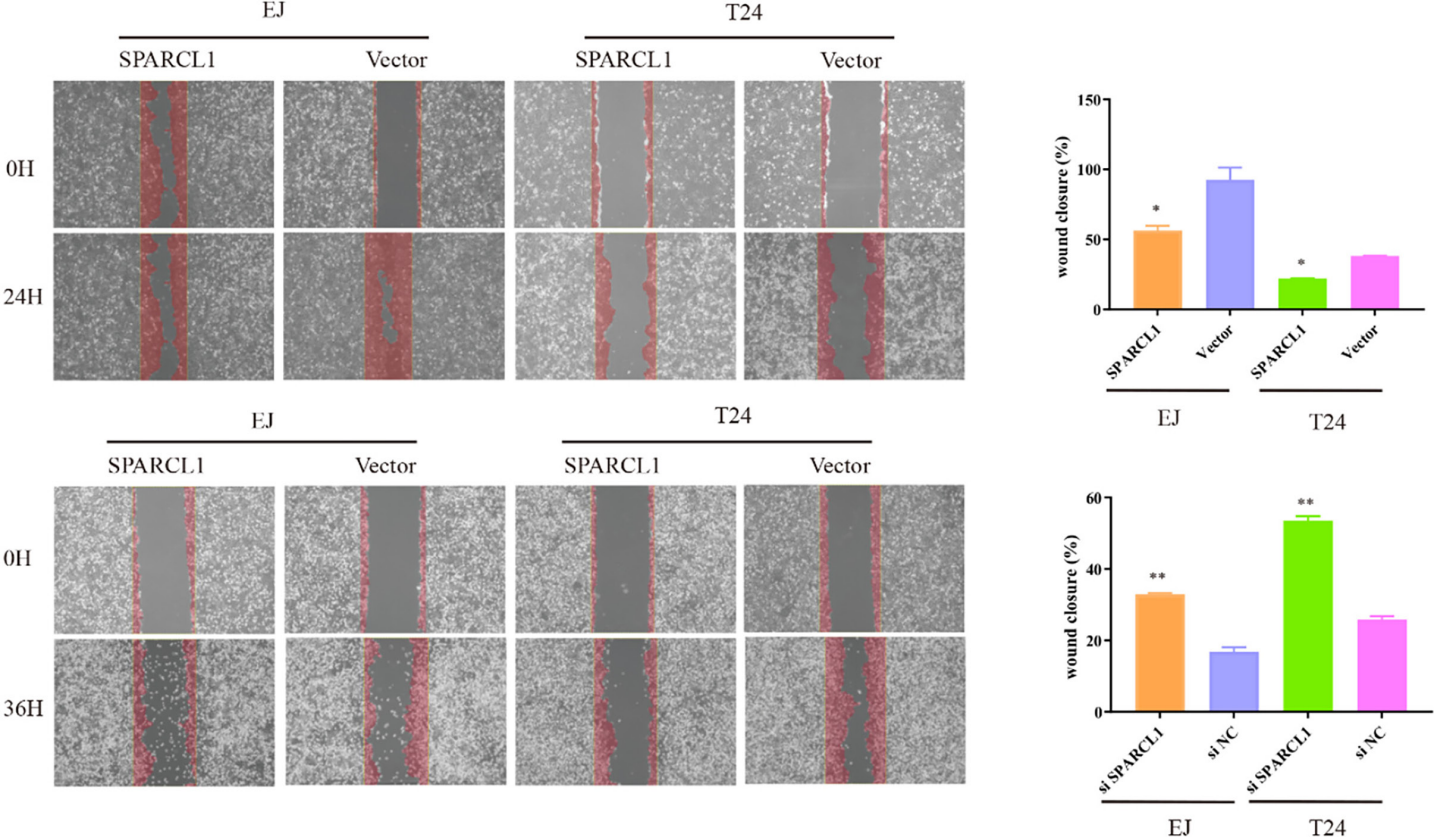
Wound healing assay showed that SPARCL1 resulted in a slower closing of scratch wounds while siSPARCL1 resulted in a faster closing of scratch wounds. Data are mean ± SD, *n* = 3. *p < 0.05, **p < 0.01 in a two‐tailed Student's *t* test.

Having observed significant inhibition of SPARCL1 in BCa, we proceeded to investigate the potential functions of SPARCL1 in vitro. To do so, we conducted a transfection experiment, introducing SPARCL1 overexpression plasmids into T24 and EJ cells. The efficacy of SPARCL1 overexpression was verified by qRT‐PCR, as depicted in Figure 3A. Subsequently, we assessed the functional impact of SPARCL1 overexpression. Utilizing a CCK‐8 assay, we discovered that the elevated expression of SPARCL1 resulted in a significant suppression of cell proliferation (Figure 3B, *p* < 0.05). Furthermore, transwell assays revealed the migration and invasion potential were notably suppressed upon SPARCL1 overexpression. These findings were consistent with the results obtained from wound healing assays (Figures 3C and 4, *p* < 0.05).

### 3.4 siSPARCL1 accelerated cell proliferation, migration and invasion in vitro

We then designed siRNAs specific to SPARCL1 and transfected them into EJ and T24 cells. qRT‐PCR analysis revealed all three si‐SPARCL1 effectively inhibited the expression of SPARCL1 in both EJ and T24 cell lines (Figure 3A). siSPARCL1‐2 was used for the subsequent experiments. CCK‐8 assays revealed that the viability of EJ and T24 was increased in siSPARCL1 subgroup compared with that in siNC subgroup (Figure 3B). In addition, the migration and invasion potential were increased in EJ and T24 cells (Figures 3C and 4, < 0.05).

### 3.5 Potential functions of SPARCL1 in BCa

**FIGURE 5:**
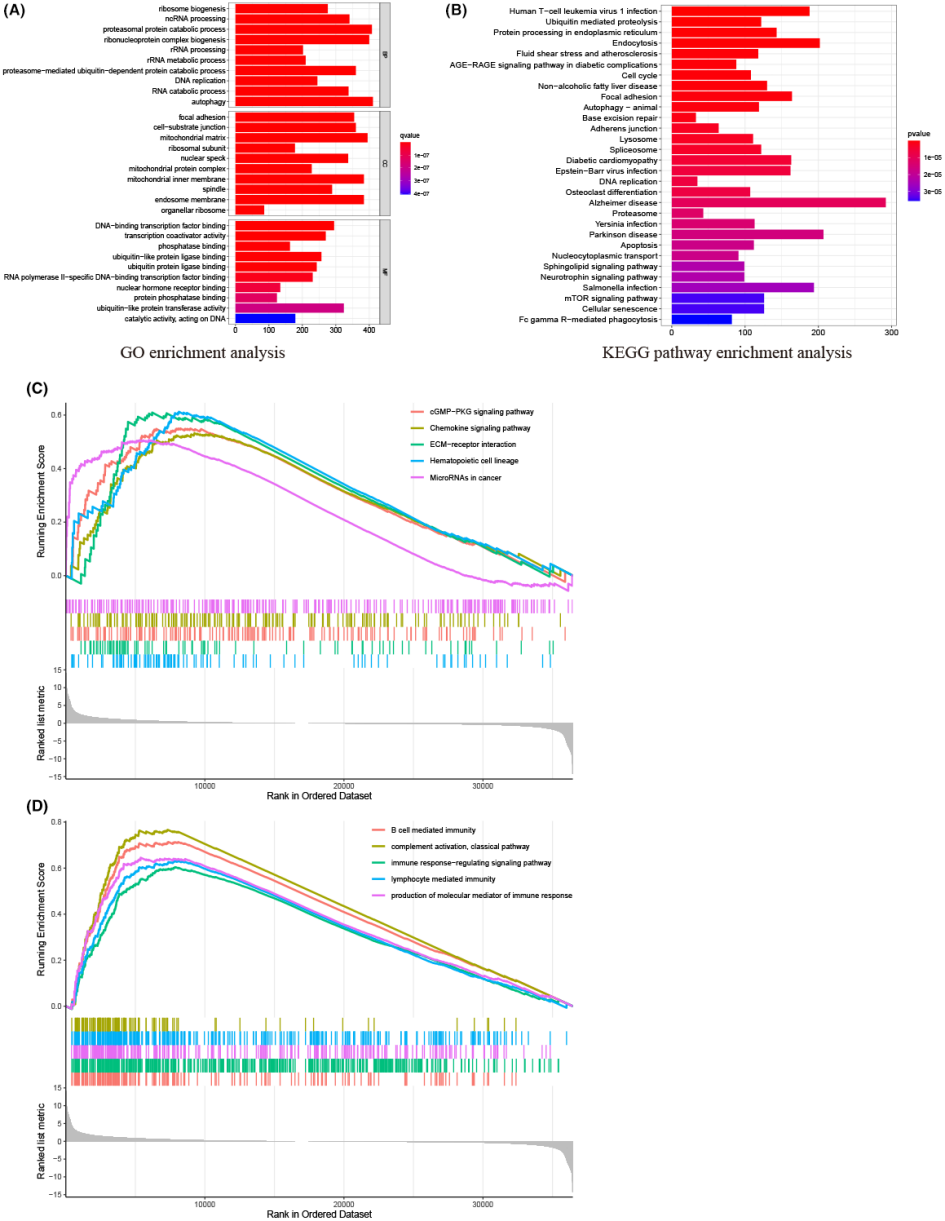
The results of GO, KEGG and GSEA analysis. (A) GO enrichment analysis. (B) KEGG pathway enrichment analysis. (C) GSEA enrichment analysis indicated SPARCL1 was enriched in several signalling pathways. (D) GSEA enrichment analysis indicated SPARCL1 was enriched in multiple immune‐related pathways.

We observed that increased levels of SPARCL1 were associated with higher histopathologic stage, T stage, N stage, and poorer OS in the clinical cohort. In contrast, in vitro experiments demonstrated that SPARCL1 inhibition led to suppressed cell proliferation, migration, and invasion. To gain insights into activated signalling pathways of SPARCL1, we conducted GO and KEGG analyses. In the GO analysis, we demonstrated high SPARCL1 expression subgroup was significantly enriched in biological processes (BP) related to ribonucleoprotein complex biogenesis, proteasomal protein catabolic process, non‐coding RNA (ncRNA) processing and ribosome biogenesis (Figure 5A). Regarding cellular components (CC), highly expressed SPARCL1 was notably associated with nuclear speck, ribosomal subunit, mitochondrial matrix, cell‐substrate junction and focal adhesion. In terms of molecular function (MF), high SPARCL1 expression showed positive correlations with ubiquitin‐like protein ligase binding, phosphatase binding, transcription coactivator activity and DNA‐binding transcription factor binding. In the KEGG analysis, SPARCL1 was found to be highly enriched in several pathways, including ubiquitin‐mediated proteolysis, endocytosis, cell cycle, base excision repair and the mTOR (mechanistic target of rapamycin) signalling pathway (Figure 5B). These findings provide insights into several potential functions of SPARCL1 in cellular processes and signalling pathways.

We conducted GSEA to further explore the potential signalling pathways associated with SPARCL1. The result found SPARCL1 was highly enriched in various pathways. These included the haematopoietic cell lineage, different extracellular matrix (ECM) receptor interaction, cGMP‐PKG signalling pathway, and chemokine signalling pathway (Figure 5C). Notably, several immune‐related pathways were significantly enriched, such as the classical pathway of complement activation, B cell‐mediated immunity, immune response‐regulating signalling pathway, production of molecular mediators of immune response, and lymphocyte‐mediated immunity (Figure 5D). Tables S1 and S2 provide detailed information on these enrichment results. These findings suggest that SPARCL1 is involved in multiple oncogenic pathways, implying its potential role in tumorigenesis in BCa. The enrichment of immune‐related pathways further highlights the potential involvement of SPARCL1 in modulating the immune response.

### 3.6 SPARCL1 expression was related to immunological features in BCa

**FIGURE 6:**
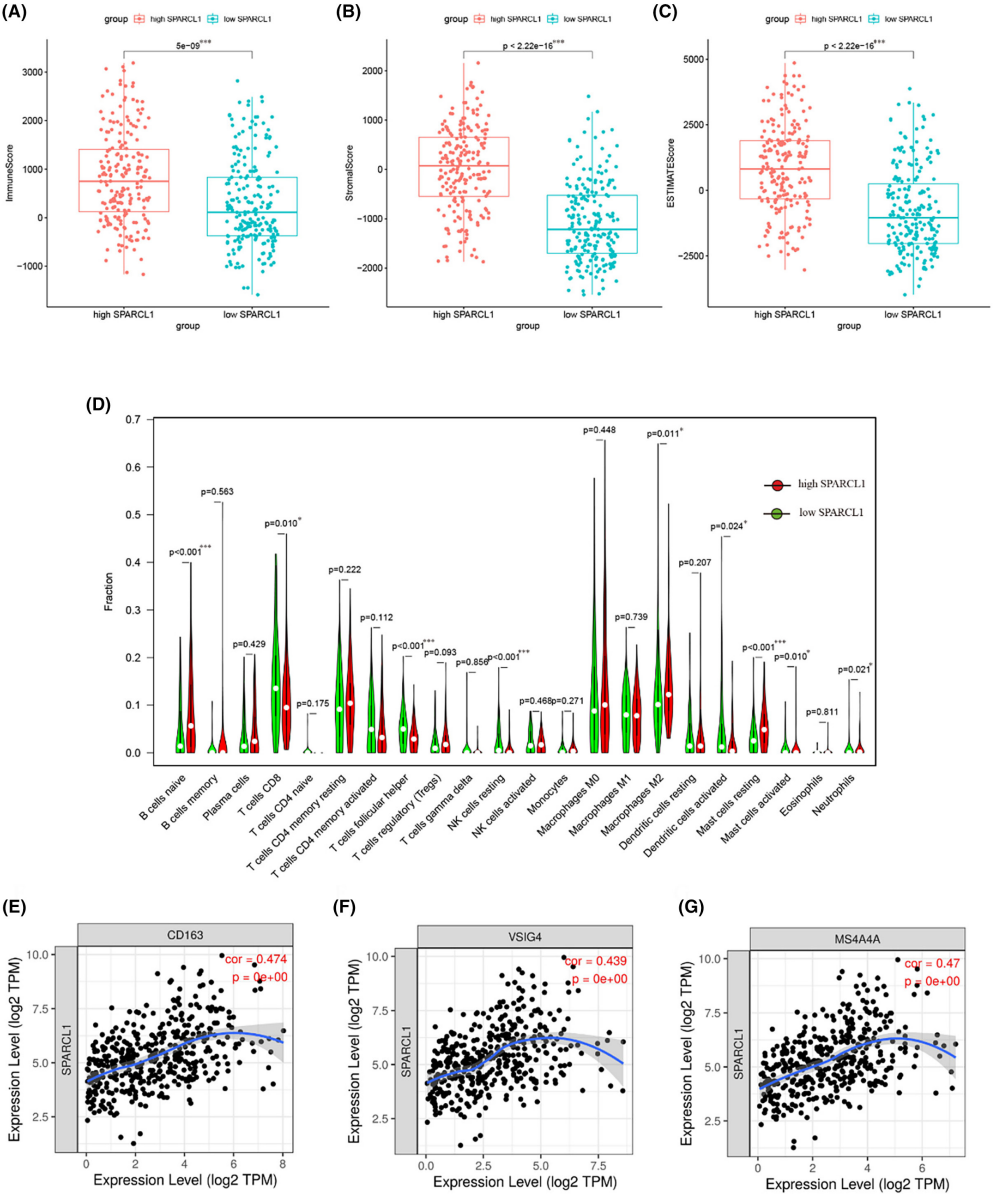
SPARCL1 expression was related to immunological features in BCa. (A–C) Highly ex‐pressed SPARCL1 was associated with elevated immune, stromal, and ESTIMATE scores. (D) The infiltration of immune cells between different SPARCL1 expression subgroups. (E–G) A moderate correlation was observed between CD163, VSIG4 and MS4A4A of M2 macrophages and SPARCL1 expression (*r* = 0.474, 0.439, 0.470, respectively). *p < 0.05, **p < 0.01 and ***p < 0.001.

Recent studies have verified the significant roles of tumour‐infiltrating lymphocytes in BCa progression. To further investigate the potential function of SPARCL1 in immune cell recruitment, we analysed the differences between ESTIMATE scores (reflecting immune and stromal cell abundance) and the expression of SPARCL1. Our analysis revealed a significant association between elevated SPARCL1 expression and increased immune, stromal and ESTIMATE scores (*p* < 0.001) (Figure 6A–C). Next, we examined the differential infiltration of immune cells among different SPARCL1 expression subgroups. The immune cell composition was calculated using the CIBERSORT algorithm, with a *p*‐value of less than 0.05 considered indicative of successful deconvolution. Our results showed that several types of immune cells exhibited differential infiltration in different SPARCL1 expression subgroups. Specifically, naive B cells, M2 macrophages, and resting mast cells were significantly elevated in the high SPARCL1 subgroup compared to the low SPARCL1 subgroup. Conversely, CD8+ T cells, follicular helper T cells, resting NK cells, and activated DC cells were increased in the low SPARCL1 group (Figure 6D). These findings indicate SPARCL1 expression might function in shaping the immune cell composition within the tumour microenvironment of BCa.

**FIGURE 7:**
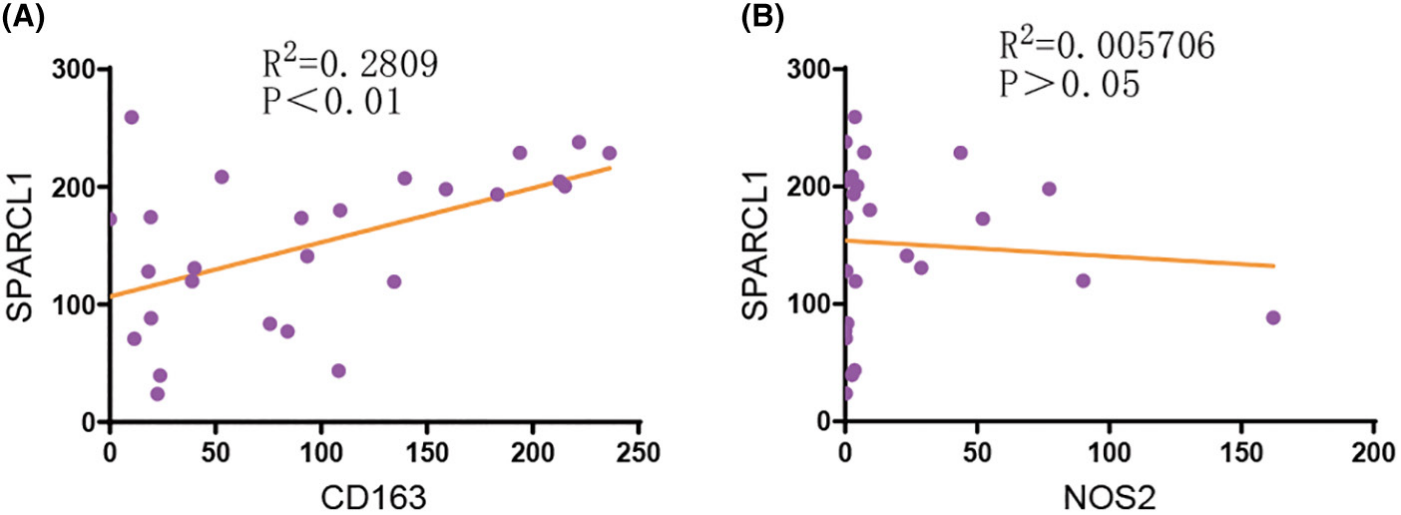
(A) The expression of CD163 was positively correlated with SPARCL1 expression (*R* ^2^ = 0.28, *p* < 0.01). (B) The expression of NOS was not associated with SPARCL1 expression (*R* ^2^ = 0.01, *p* > 0.05).

We then investigated whether SPARCL1 was correlated with infiltration level of immune cells. Our study demonstrated highly expressed SPARCL1 was associated with increased infiltration of immune cells, specifically CD163, VSIG4 and MS4A4A (*r* = 0.474, 0.439, 0.470, respectively, *p* < 0.001) (Figure 6E–G). The IHC analysis based on clinical specimens verified the expression of CD163 (the specific biomarker of M2 macrophages) was positively correlated with SPARCL1 expression (*R* ^2^ = 0.28, *p* < 0.01). The expression of NOS2 (the specific biomarker of M1 macrophages) was not associated with SPARCL1 expression (*R* ^2^ = 0.01, *p* > 0.05) (Figure 7A,B). Furthermore, the expression of CXCR5, a marker of follicular helper T cells, was also positively correlated with SPARCL1 expression (*r* = 0.486, *p* < 0.001) (Figure S1). Additionally, immune markers associated with NK cells (FCGR3A, FCGR3B and NCAM1) and CD8+ T cells (CD8A and CD8B) showed associations with SPARCL1 mRNA levels (Figure S1).

Collectively, these findings provide further evidence suggesting that SPARCL1 might function during recruitment of immune cells within tumour microenvironment.

### 3.7 SPARCL1 expression was related to TMB, activities of immune activation processes, TIDE and immune checkpoint expression in BCa

**FIGURE 8:**
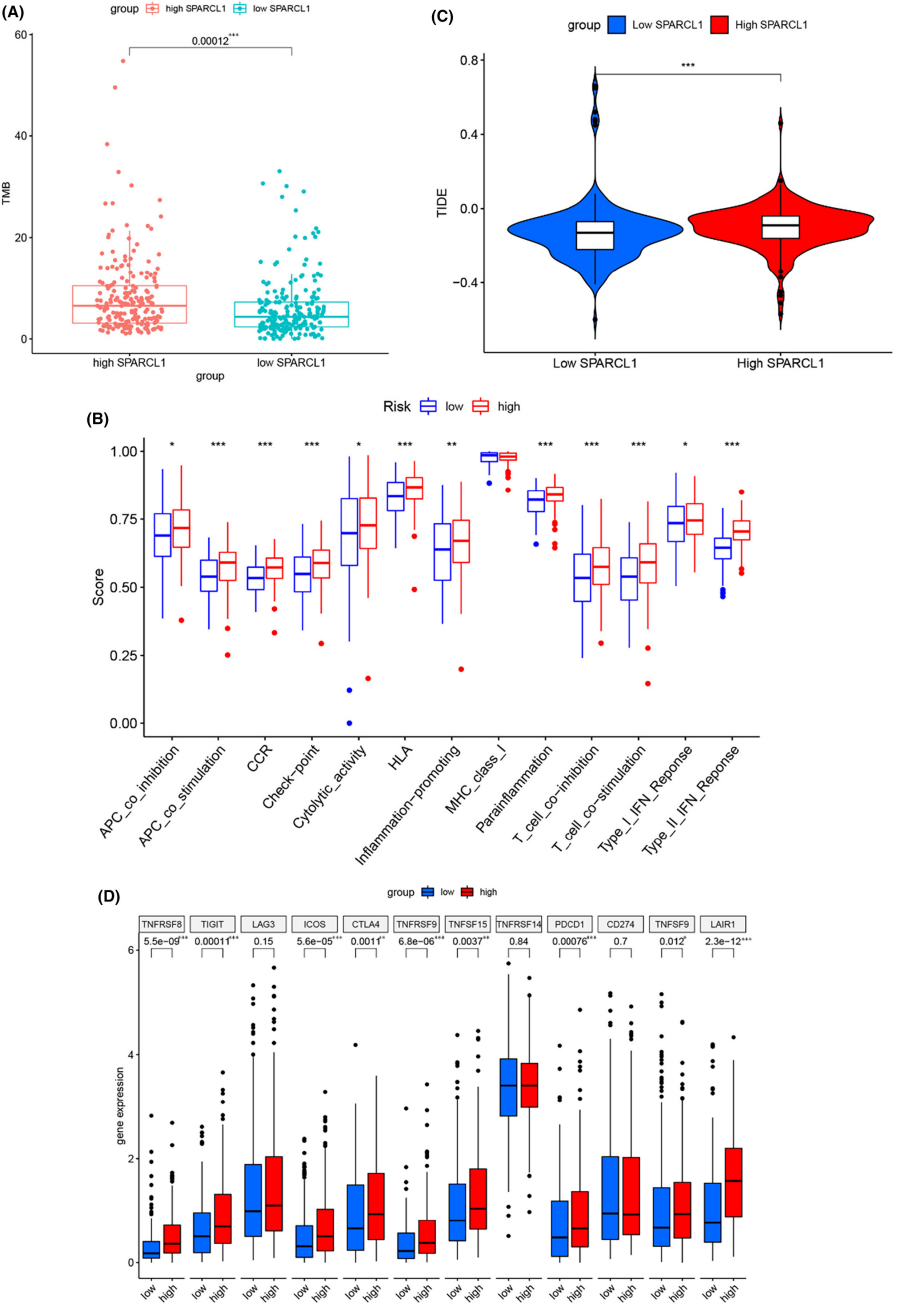
SPARCL1 expression was related to TMB, activities of immune activation processes, TIDE, and immune checkpoint expression in BCa. (A) Patients with highly expressed SPARCL1 contained a higher TMB. (B) Most of the critical immune activation processes, such as CCR, check‐point, HLA, T cell co‐inhibition and T cell co‐stimulation, were activated in highly expressed SPARCL1 subgroup. (C) TIDE score was higher in highly expressed SPARCL1 subgroup. (D) TNFRSF8, TIGIT, CTLA4, LAIR1, ICOS, PDCD1 were elevated in high SPARCL1 expression group. *p < 0.05, **p < 0.01 and ***p < 0.001.

We then figured out potential relationship between SPARCL1 and efficacy of immune checkpoint inhibitors (ICIs). Since TMB is an effective marker for predicting ICI response,^23^ we evaluated the impact of SPARCL1 expression on TMB. Our analysis revealed patients in the high SPARCL1 expression subgroup contained higher TMB (*p* < 0.001) (Figure 8A). To explore the potential immune processes associated with SPARCL1, we employed ssGSEA to assess immune pathway activities. We observed that crucial immune activation processes, including chemokine receptor (CCR) signalling, immune checkpoints, human leukocyte antigen (HLA) pathways, and T cell co‐inhibition, were more prominently activated in the high SPARCL1 expression subgroup (Figure 8B).

Next, we utilized the TIDE score, which combines dysfunction and exclusion scores, to evaluate immune escape and immunotherapeutic response. Since the TIDE score has the potential to predict immunotherapeutic response, we calculated the TIDE score for every BCa sample. Notably, we demonstrated that the TIDE score was strongly elevated in highly expressed SPARCL1 subgroup (*p* < 0.001) (Figure 8C). Although there was no statistical difference observed in the expression of PD‐L1 (also known as CD274) between different SPARCL1 expression subgroups, several other immune checkpoint molecules (TNFRSF8, TIGIT, CTLA4, LAIR1, ICOS, PDCD1) were elevated in the high SPARCL1 expression subgroup (Figure 8D). These results strongly support the prognostic implications of SPARCL1 in immunotherapy. While these findings provide valuable insights into the immunotherapeutic response, it is important to note that further studies exploring underlying mechanisms and larger sample sizes are warranted to fully understand the implications of SPARCL1 in immunotherapy.

### 3.8 SPARCL1 expression was related to chemotherapeutic sensitivity

**FIGURE 9:**
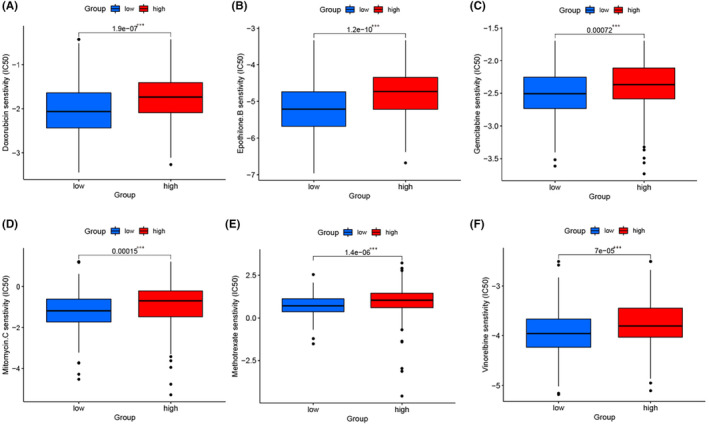
SPARCL1 expression was related to chemotherapeutic sensitivity. (A) Patients with highly expressed SPARCL1 were found to have a better response to doxorubicin. (B) Patients with highly expressed SPARCL1 were found to have a better response to epothilone. (C) Patients with highly expressed SPARCL1 were found to have a better response to gemcitabine. (D) Patients with highly expressed SPARCL1 were found to have a better response to mitomycin.C. (E) Patients with highly expressed SPARCL1 were found to have a better response to methotrexate. (F) Patients with highly expressed SPARCL1 were found to have a better response to vinorelbine. ***p < 0.001.

To assess the drug sensitivity of BCa patients in two SPARCL1 subgroups, we calculated the IC50 values of several chemotherapeutic drugs. Notably, the IC50 values of doxorubicin, epothilone B, vinorelbine, mitomycin C, methotrexate and gemcitabine were found to be higher in the highly expressed SPARCL1 subgroup (*p* < 0.001) (Figure 9). Among these drugs, mitomycin C and gemcitabine are critical for intravesical chemotherapy, while methotrexate, doxorubicin, and gemcitabine are indispensable for systemic chemotherapy. These findings indicate that BCa patients with high SPARCL1 expression may derive greater benefit from both intravesical and systemic chemotherapy. The higher IC50 values observed in the highly expressed SPARCL1 subgroup suggest that these patients may require higher drug concentrations or alternative treatment strategies to achieve optimal therapeutic efficacy. This information can assist in guiding treatment decisions and personalized approaches for BCa patients based on their SPARCL1 expression levels.

## 4 DISCUSSION

BCa is known for its heterogeneity, requiring personalized management due to variations in invasion, recurrence, and drug resistance among BCa cells. These factors contribute to the relatively short median survival of BCa patients. Despite extensive research efforts to understand the molecular characteristics of BCa, only a minority of patients exhibit long‐term responses to chemotherapy or immunotherapy, highlighting the urgent need for effective biomarkers. SPARCL1, a secreted glycoprotein, has been frequently found to be inhibited in various cancer types. Convincing evidence has highlighted the critical functions of SPARCL1 in muscle damage repair and inhibition of DNA synthesis.^14^, ^24^ However, there is a paucity of studies investigating the role of SPARCL1 in BCa.

Our findings confirmed the downregulation of SPARCL1 in several human tumours, including BCa, BRCA, COAD, HNSC, KIRP, LUAD, LUSC, PRAD, READ, THCA, UCEC and STAD. Conversely, SPARCL1 was found to be upregulated in KICH, KIRC, LIHC and CHOL. These observations underscore the significant roles of SPARCL1 in inhibiting tumorigenesis. Our results align with previous studies.^25^ SPARCL1 has been extensively investigated in gastrointestinal tumours. Notably, it has been established that SPARCL1 is reduced in colorectal cancer.^15^ In colorectal cancer, upregulation of SPARCL1 has been associated with favourable survival outcomes, while downregulation of SPARCL1 is observed in more aggressive cases.^15^, ^26^ Moreover, SPARCL1 has been shown to impede motility in colorectal carcinoma and is linked to tumour stage and metastasis.^26^, ^27^ In oesophageal cancer, SPARCL1 was verified to inhibit cell proliferation, invasion, and glycolysis.^28^ While some researches have confirmed a tumour‐suppressive role of SPARCL1 across various cancer types,^25^, ^29^, ^30^ it has also been demonstrated to promote cancer progression in specific cancers, indicating diverse functions of SPARCL1 depending on the type of cancer. For instance, SPARCL1 is highly expressed in hepatocellular carcinoma, yet its upregulation restrains cell proliferation in this context.^31^, ^32^ The significant heterogeneity observed among tumours is a prominent feature. Therefore, we hypothesize that SPARCL1 may exert distinct functions in promoting or inhibiting the carcinogenesis process depending on the specific ECM environments encountered.

We conducted a comprehensive analysis of SPARCL1 in BCa to gain a deeper understanding of its role in the disease. Our findings revealed that although SPARCL1 was generally down‐regulated in BCa, clinical information indicated SPARCL1 may function as a risk gene in BCa tumorigenesis. Specifically, we observed an upregulation of SPARCL1 expression as the BCa stage advanced, indicating a potential correlation between SPARCL1 transcriptional levels and disease progression. Furthermore, higher expressed SPARCL1 was related to an increased likelihood of higher T stage, suggesting its involvement in tumour growth and invasion. Meanwhile, a significant association was observed between SPARCL1 expression and lymphatic metastasis. Interestingly, the results of cytological experiments yielded opposite findings, as SPARCL1 was verified to inhibit cell proliferation, migration, and invasion in vitro. To further figure out the underlying mechanism behind this inconsistency, we performed GO, KEGG, and GSEA analysis. Notably, our results demonstrated SPARCL1 was enriched in multiple immune‐related pathways, including the classical pathway of complement activation, B cell‐mediated immunity, immune response‐regulating signalling pathway, production of molecular mediators of immune response, and immune response‐regulating signalling pathway. These findings strongly suggest that SPARCL1 plays a crucial role in the recruitment of immune cells and is involved in immune‐related processes in BCa. Taken together, our comprehensive analysis highlights the complex and multifaceted role of SPARCL1 in BCa. While its downregulation in clinical samples may indicate a potential risk gene during BCa tumorigenesis, in vitro experiments demonstrate its inhibitory effects on cancer cell behaviour. The enrichment of immune‐related pathways further emphasizes the involvement of SPARCL1 in immune cell recruitment and immune responses in BCa.

To investigate the association between SPARCL1 and immunological features, we conducted a detailed analysis. Our findings revealed several significant correlations between SPARCL1 expression and various immune‐related parameters. Specifically, a positive association with SPARCL1 expression and immune and stromal scores was verified. Furthermore, SPARCL1 showed a positive association with the abundance of naive B cells, M2 macrophages, and resting mast cells. Conversely, SPARCL1 exhibited a negative correlation with the presence of CD8+ T cells, follicular helper T cells, resting NK cells, and activated DC cells. These findings indicate that SPARCL1 serves as a critical role in shaping immune landscape within the tumour microenvironment. To further support our results, we examined the correlation between SPARCL1 expression and specific biomarkers of M2 macrophages, such as CD163, VSIG4 and MS4A4A. We found a moderate correlation between these markers' expression and SPARCL1, providing additional evidence for the potential involvement of SPARCL1 in macrophage recruitment and polarization. Taken together, our results indicate that SPARCL1 may have important functions in modulating immune responses in tumour microenvironment. The positive associations with immune and stromal scores, as well as the abundance of specific immune cell subsets, suggest that SPARCL1 could influence immune cell recruitment and activation. The correlation with markers of M2 macrophages further supports the notion that SPARCL1 may be involved in macrophage polarization.

Macrophages represent a significant proportion of non‐cancerous cells within the tumour microenvironment. Previous studies have highlighted the crucial role of tumour‐associated macrophages (TAMs) in carcinogenesis.^33^, ^34^ However, the specific association between SPARCL1 and macrophages remains poorly understood. A prior investigation demonstrated that SPARCL1 recruits macrophages by activating the WNT/β‐catenin signalling pathway in osteosarcoma.^30^ Similarly, in gastrointestinal stromal tumours, SPARCL1 was found to downregulate M2 polarization and suppress macrophage recruitment by inhibiting the phosphorylation of p65.^35^ Despite these findings, the precise impact of SPARCL1 on macrophage polarization in the context of BCa remains unclear.

Macrophages exhibit extreme heterogeneity and can be influenced by various factors. They are commonly categorized into two phenotypes, M1 and M2, in response to external stimuli.^36^ While both types are associated with inflammatory responses, their functions differ significantly. M2 macrophages are primarily involved in the process of carcinogenesis, whereas M1 macrophages play a role in anti‐metastatic mechanisms in cancer cells.^34^, ^37^, ^38^ Evidence has shown that cancer cells exploit M2 macrophages to invade tissues and evade immune responses.^37^ TAMs have been found to express multiple cytokines, such as epidermal growth factor, platelet‐derived growth factor, and transforming growth factor‐β, which promote the proliferation and migration of cancer cells.^39^ TAMs have also been shown to express PD‐L1 and serve as significant mediators of tissue homeostasis.^40^ Treatment of TAMs with PD‐L1 antibodies has been shown to enhance macrophage phagocytosis, reduce tumour growth, and improve survival in mouse models of cancer. This suggests that PD‐L1 antibody therapy may elicit a better response in patients with higher infiltration of TAMs within tumours.^41^, ^42^ Our study observed highly expressed SPARCL1 was related to elevated TMB, TIDE score, and multiple immune checkpoints (TNFRSF8, TIGIT, CTLA4, LAIR1, ICOS, PDCD1). These findings strongly support the prognostic implications of SPARCL1 in immunotherapy. The increased expression of immune checkpoints and the higher TMB and TIDE scores in the high SPARCL1 expression group suggest that these patients may be more responsive to immunotherapy interventions. Overall, our results highlight the potential of SPARCL1 as a valuable indicator in context of immunotherapy. The elevated immune checkpoints and association with TMB and TIDE scores further suggest that SPARCL1 might function as a valuable prognostic biomarker for predicting immunotherapy efficiency.

We evaluated the impact of SPARCL1 expression on the sensitivity to various canonical chemotherapeutic drugs. We determined the IC50 values for several commonly used chemotherapeutic agents, including doxorubicin, epothilone B, vinorelbine, mitomycin C, methotrexate and gemcitabine, in different subgroups based on SPARCL1 expression levels. Interestingly, we observed that the high SPARCL1 expression subgroup displayed higher IC50 values for these drugs. Of particular relevance, mitomycin C and gemcitabine are critical chemotherapeutic drugs used in intravesical chemotherapy for BCa. Additionally, methotrexate, doxorubicin, and gemcitabine are indispensable agents in systemic chemotherapy for various cancer types, including BCa.^43^, ^44^, ^45^, ^46^ Our findings suggest that patients with high SPARCL1 expression may derive greater benefit from both intravesical and systemic chemotherapy regimens due to the higher IC50 values observed for these drugs in this subgroup. These results emphasize the potential clinical implications of SPARCL1 expression levels in guiding treatment decisions for BCa patients. Specifically, patients with higher SPARCL1 expression may require carefully tailored therapeutic strategies, considering the reduced sensitivity to certain chemotherapeutic agents.

This study is subject to several limitations that should be acknowledged. First, our study cohort was derived from public datasets, which introduces inherent limitations such as selection bias and potential tumour heterogeneity, thereby affecting the robustness of our findings. Additionally, our manuscript primarily focused on demonstrating the immune interactions and potential activated signalling pathways associated with SPARCL1 expression. However, further investigations are essential for the underlying mechanisms involved. While our study revealed a reduction in SPARCL1 expression in BCa, survival analysis indicated a significant association with highly expressed SPARCL1 and poor prognosis. The elevated ESTIMATE scores, abundance of naive B cells, M2 macrophages, resting mast cells, increased TMB and TIDE score, as well as the upregulation of multiple immune checkpoints in the high SPARCL1 expression subgroup, led us to speculate that SPARCL1 may play an indispensable role in shaping the immune microenvironment, which could potentially counteract its anti‐cancer effects.

## 5 CONCLUSIONS

In summary, our study provides evidence SPARCL1 is downregulated in various cancer types. Elevated SPARCL1 expression was linked with advanced tumour stage, increased likelihood of lymphatic metastasis, and poorer prognosis in our clinical cohort. In vitro experiments demonstrated that SPARCL1 inhibition led to accelerated cell proliferation, migration and invasion. In addition, we revealed a significant association with SPARCL1 expression and immunological features in BCa. This included an association with higher TMB, increased activity of immune activation processes, TIDE score, expression of immune checkpoints, and enhanced sensitivity to chemotherapy. In summary, our study highlights the multifaceted function of SPARCL1 in cancer biology. It not only provides insights into its impact on tumour behaviour and clinical outcomes but also suggests its involvement in immune‐related processes. Further investigations are warranted to unravel the precise mechanisms underlying the functions of SPARCL1 and to validate its utility as a prognostic marker and therapeutic target in various cancer contexts.

## AUTHOR CONTRIBUTIONS

**Changjiu Li:** Conceptualization (lead); data curation (lead); methodology (lead); writing – original draft (lead). **Hui Yuan:** Data curation (equal); writing – review and editing (equal). **Jun Chen:** Data curation (equal); resources (equal). **Kun Shang:** Investigation (equal); visualization (equal). **Huadong He:** Supervision (lead); validation (lead); writing – review and editing (lead).

## CONFLICT OF INTEREST STATEMENT

The authors declare no conflict of interest.

## Supporting information


Figure S1.

Figure S2.

Table S1.

Table S2.

## Data Availability

The data of this study are available from the TCGA (https://portal.gdc.cancer.gov/) database, TIMER2.0 database (http://timer.cistrome.org/), and the TIMER database (https://cistrome.shinyapps.io/timer/).
