# Supplementary material for: The oncogenic functions of SPARCL1 in bladder cancer
Source: J Cell Mol Med. 2024 Nov 15;28(22):e70196. doi: 10.1111/jcmm.70196 (PMC11567778; doi:10.1111/jcmm.70196)
Supplement: Supplementary file 1 — Figure S1. [file JCMM-28-e70196-s004.docx]

## Supplementary Figure
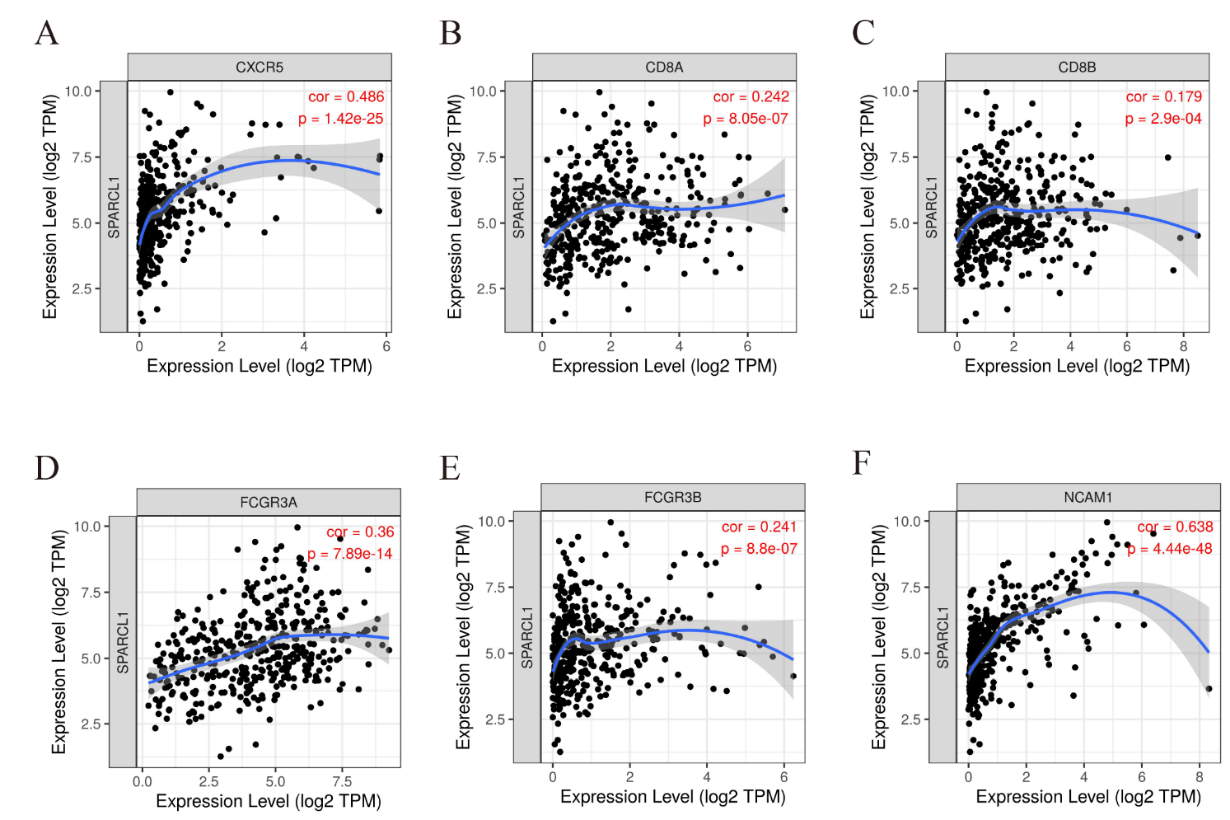


**Supplementary Figure 1.** The correlation of SPARCL1 and CXCR5, CD8A, CD8B, FCGR3A, FCGR3B, and NCAM1.
