# Supplementary material for: The oncogenic functions of SPARCL1 in bladder cancer
Source: J Cell Mol Med. 2024 Nov 15;28(22):e70196. doi: 10.1111/jcmm.70196 (PMC11567778; doi:10.1111/jcmm.70196)
Supplement: Supplementary file 2 — Figure S2. [file JCMM-28-e70196-s001.docx]

## Supplementary Figure


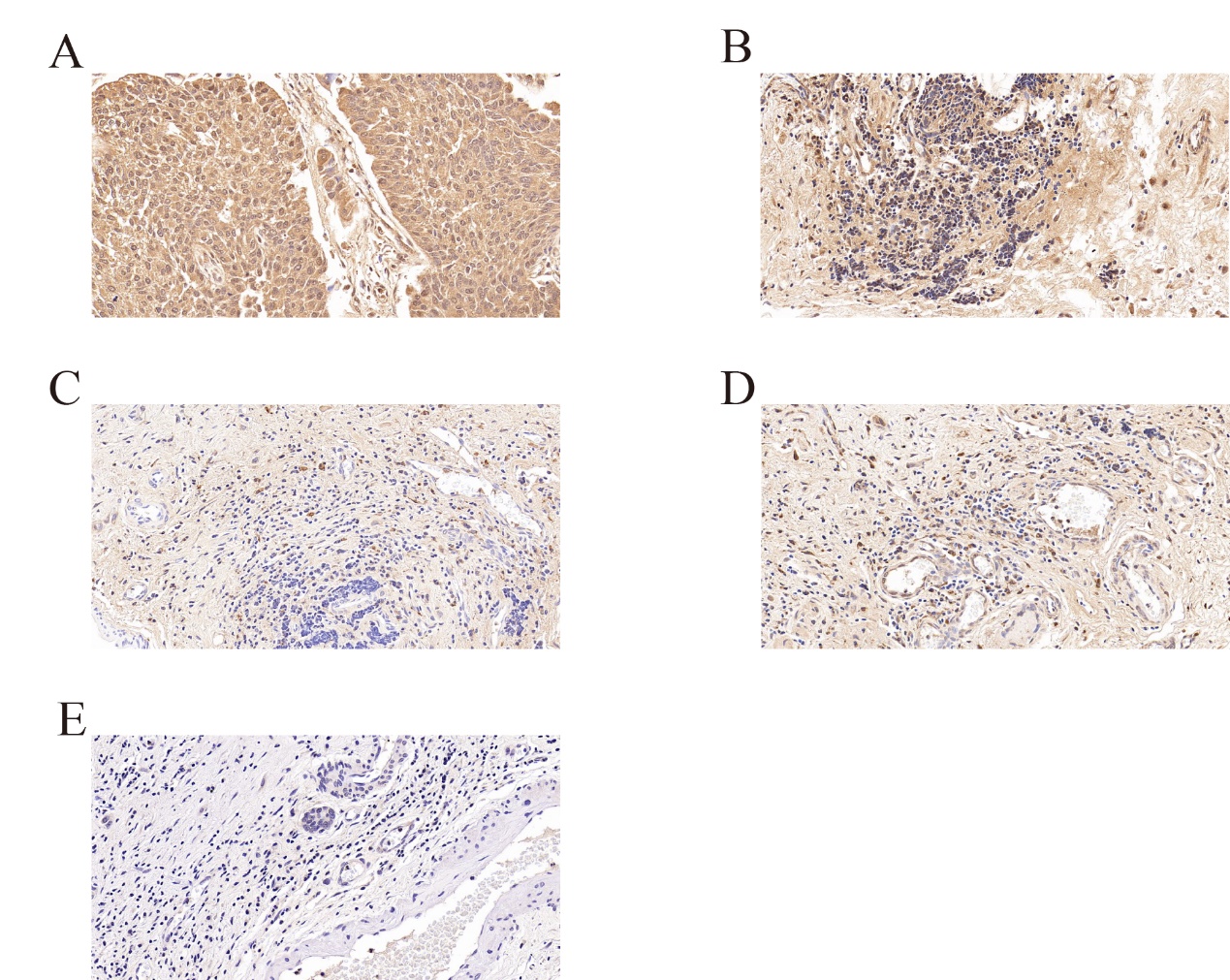


**Supplementary Figure 2.** (A) The protein level of SPARCL1 in normal tissues. (B) The protein level of SPARCL1 in T4G3 BCa tissues. (C) The protein level of SPARCL1 in T2G1 BCa tissues. (D) The protein level of SPARCL1 in lymphatic metastatic BCa tissues. (E) The protein level of SPARCL1 in non-lymphatic metastatic BCa tissues.
